# Effects of smoking and smoking cessation on human serum metabolite profile: results from the KORA cohort study

**DOI:** 10.1186/1741-7015-11-60

**Published:** 2013-03-04

**Authors:** Tao Xu, Christina Holzapfel, Xiao Dong, Erik Bader, Zhonghao Yu, Cornelia Prehn, Katrin Perstorfer, Marta Jaremek, Werner Roemisch-Margl, Wolfgang Rathmann, Yixue Li, H -Erich Wichmann, Henri Wallaschofski, Karl H Ladwig, Fabian Theis, Karsten Suhre, Jerzy Adamski, Thomas Illig, Annette Peters, Rui Wang-Sattler

**Affiliations:** 1 Research Unit of Molecular Epidemiology, Helmholtz Zentrum München, Ingolstädter Landstraße 1, Neuherberg, 85764, Germany; 2 Else Kroener-Fresenius-Center for Nutritional Medicine, University Hospital, Klinikum rechts der Isar, Technische Universität München, Ismaninger Straße 22, Munich, 81675, Germany; 3 Key Laboratory of Systems Biology, Shanghai Institutes for Biological Sciences, Chinese Academy of Sciences, Yue Yang Road 320, Shanghai, 200031, China; 4 University of Chinese Academy of Sciences, Yuquanlu 19A, Beijing, 100049, China; 5 Institute of Experimental Genetics, Genome Analysis Center, Helmholtz Zentrum München, Ingolstädter Landstraße 1, Neuherberg, 85764, Germany; 6 Institute of Bioinformatics and Systems Biology, Helmholtz Zentrum München, Ingolstädter Landstraße 1, Neuherberg, 85764, Germany; 7 Institute of Biometrics and Epidemiology, German Diabetes Center, Leibniz Center for Diabetes Research at Heinrich Heine University, Moorenstr. 5, Düsseldorf, 40225, Germany; 8 Institute of Epidemiology I, Helmholtz Zentrum München, Ingolstädter Landstraße 1, Neuherberg, 85764, Germany; 9 Institute of Medical Informatics, Biometry and Epidemiology, Ludwig-Maximilians-Universität, Marchioninistr. 15, Munich, 81377, Germany; 10 Institute of Clinical Chemistry and Laboratory Medicine, University Medicine Greifswald; Ernst-Moritz-Arndt University, Ferdinand-Sauerbruch-Straße, Greifswald, 17475, Germany; 11 Institute of Epidemiology II, Helmholtz Zentrum München, Ingolstädter Landstraße 1, Neuherberg, 85764, Germany; 12 Department of Psychosomatic Medicine and Psychotherapy, University Hospital, Klinikum rechts der Isar, Technische Universität München, Ismaninger Straße 22, Munich, 81675, Germany; 13 Department of Mathematics, Technische Universität München, Boltzmannstraße 3, Garching, 85748, Germany; 14 Faculty of Biology, Ludwig-Maximilians-Universität, Großhaderner Straße 2-4, Planegg-Martinsried, 82152, Germany; 15 Department of Physiology and Biophysics, Weill Cornell Medical College in Qatar (WCMC-Q), PO Box 24144, Doha, Qatar; 16 Chair of Experimental Genetics, Technische Universität München, Ingolstädter Landstraße 1, Neuherberg, 85764, Germany; 17 Hannover Unified Biobank, Hannover Medical School, Carl-Neuberg-Straße 1, Hannover, 30625, Germany; 18 Harvard School of Public Health, Department of Environmental Health, 677 Huntington Avenue, Boston, 02115, USA

**Keywords:** metabolic network, metabolomics, molecular epidemiology, smoking, smoking cessation

## Abstract

**Background:**

Metabolomics helps to identify links between environmental exposures and intermediate biomarkers of disturbed pathways. We previously reported variations in phosphatidylcholines in male smokers compared with non-smokers in a cross-sectional pilot study with a small sample size, but knowledge of the reversibility of smoking effects on metabolite profiles is limited. Here, we extend our metabolomics study with a large prospective study including female smokers and quitters.

**Methods:**

Using targeted metabolomics approach, we quantified 140 metabolite concentrations for 1,241 fasting serum samples in the population-based Cooperative Health Research in the Region of Augsburg (KORA) human cohort at two time points: baseline survey conducted between 1999 and 2001 and follow-up after seven years. Metabolite profiles were compared among groups of current smokers, former smokers and never smokers, and were further assessed for their reversibility after smoking cessation. Changes in metabolite concentrations from baseline to the follow-up were investigated in a longitudinal analysis comparing current smokers, never smokers and smoking quitters, who were current smokers at baseline but former smokers by the time of follow-up. In addition, we constructed protein-metabolite networks with smoking-related genes and metabolites.

**Results:**

We identified 21 smoking-related metabolites in the baseline investigation (18 in men and six in women, with three overlaps) enriched in amino acid and lipid pathways, which were significantly different between current smokers and never smokers. Moreover, 19 out of the 21 metabolites were found to be reversible in former smokers. In the follow-up study, 13 reversible metabolites in men were measured, of which 10 were confirmed to be reversible in male quitters. Protein-metabolite networks are proposed to explain the consistent reversibility of smoking effects on metabolites.

**Conclusions:**

We showed that smoking-related changes in human serum metabolites are reversible after smoking cessation, consistent with the known cardiovascular risk reduction. The metabolites identified may serve as potential biomarkers to evaluate the status of smoking cessation and characterize smoking-related diseases.

## Background

Smoking is responsible for 90% of all lung cancers, accounts for 25% of cancer deaths worldwide [1-3] and is a significant risk factor for cardiovascular disease (CVD) [4-7]. The benefits of smoking cessation are remarkable. Risk of CVD is reduced in former smokers (FS) compared with current smokers (CS) [8-10]; mortality and future cardiac events both decline in FS [11,12]. Nevertheless, for cancers, especially for adenocarcinoma, the risk remains high in FS compared with never smokers (NS) [13,14]. Studies have made attempts to find the molecular basis for the influence of smoking and smoking cessation on cardiovascular risks. For instance, smoking is associated with the increase of several CVD-related inflammatory markers, for example, c-reactive protein and fibrinogen [15-17], and smoking cessation could largely reduce the level of these markers [18]. However, there is also evidence that other molecular changes associated with smoking are permanent, for example, loss of heterozygosity and hypermethylation in the promoter regions of cancer-related genes [19-23].

The metabolomics approach provides a functional readout of activities located downstream of the gene expression level that are more closely related to the physiological status [24] and, thus, may be particularly useful for the study of environmental influences, namely the 'exposome' [25]. Studying a strong environmental factor, for example a lifestyle-related exposure to smoking, may be considered a very powerful approach for understanding the links between environmental exposure and the metabolome. In human lung epithelial cells, it has been shown that metabolite concentration changes in various pathways, for example, the urea cycle and polyamine metabolism and lipid metabolism under smoke exposure [26]. In a pilot study with 283 male participants from the Cooperative Research in the Region of Augsburg (KORA) F3 in Germany, we have shown that levels of diacyl-phosphatidylcholines (PCs) were higher in 28 CS compared with 101 NS, except for acyl-alkyl-PCs [1]. The reduced ratios of acyl-alkyl-to diacyl-PCs in CS may be regulated by the enzyme alkyl-dihydroxyacetone phosphate in both ether lipid and glycerophospholipid pathways [1]. However, little has been reported about the reversibility of the metabolite profile upon smoking cessation, which is important for comprehensive understanding of smoking effects. It is also known that metabolite profile is different between men and women [25], but whether lifestyle factors such as smoking may induce different metabolite patterns in men and women is still unknown.

In this study, we analyzed the association between smoking and the concentration of metabolites in 1,241 serum samples from the KORA baseline survey 4 (S4) and follow-up (F4) study, aiming to extend the knowledge of smoking-associated metabolites beyond our pilot study by including female CS at two time points over seven years, to investigate whether smoking-associated changes in metabolite profile are reversible after smoking cessation, and to provide insights into the pathophysiological consequences of smoking in protein-metabolite networks.

## Methods

### Ethics statement

Written informed consent was obtained from KORA S4 and F4 participants. The KORA study was approved by the ethics committee of the Bavarian Medical Association in Munich, Germany.

### Study population

The KORA surveys are population-based studies conducted in the Region of Augsburg in Germany [27,28]. Four surveys were conducted with 18,079 participants recruited from 1984 to 2001. The S4 consists of 4,261 individuals (25 to 74 years old) examined from 1999 to 2001. From 2006 to 2008, 3,080 participants (with an age range of 32 to 81 years) took part in the F4 survey. Each participant completed a lifestyle questionnaire providing information on a number of parameters including smoking status (current, former, never). Serum samples for metabolomics analysis were collected in parallel in the KORA S4 and F4 survey as described elsewhere [29-31].

For metabolite profiles, serum samples from 1,614 people aged 55 to 74 years old were available [29]. Participants with non-fasting status (N = 216) or missing values (N = 22) were excluded from the analysis. We further excluded 145 people in KORA S4 and 116 people in the longitudinal data of KORA S4 → F4, whose spouses were CS, to rule out passive smoking effects. Furthermore, metabolite concentrations of serum samples from 1,036 participants were measured in both KORA S4 and F4.

### Metabolite measurements

Liquid handling of serum samples (10 μl) was performed with Hamilton star robot (Hamilton Bonaduz AG, Bonaduz, Switzerland) and prepared for quantification using the Absolute*IDQ *P180 and P150 kits (BIOCRATES Life Science AG, Innsbruck, Austria) for the KORA S4 and F4 surveys, respectively. This allowed simultaneous quantification of 188 or 163 metabolites using liquid chromatography and flow injection analysis mass spectrometry as described previously [32,33]. The complete analytical process was monitored by quality control steps, reference samples and the MetIQ software package, which is an integral part of the Absolute *IDQ *kit.

Because the two datasets were generated by different platforms, different quality control processes were introduced. The metabolite data quality control procedure for the KORA S4 samples was described in our recently published work [29]. There were 140 metabolites that passed the two quality controls: one hexose, 21 amino acids, eight biogenic amines, 21 acylcarnitines, 13 sphingomyelins (SMs), eight lysoPCs, 33 diacyl-PCs (PC aa Cx:y) and 35 acyl-alkyl-PCs (PC ae Cx:y). Lipid side chain composition is abbreviated as Cx:y, where × denotes the number of carbons in the side chain and y the number of double bonds. The precise position of the double bonds and the distribution of the carbon atoms in different fatty acid side chains cannot be determined with this technology. Concentrations of all analyzed metabolites are reported in μmol/L (μM). The data cleaning procedure for the KORA F4 samples has previously been described in detail [24,30]. In total, 121 metabolites were measured in both S4 and F4, and used for the prospective study.

### Statistical analysis

**Figure 1:**
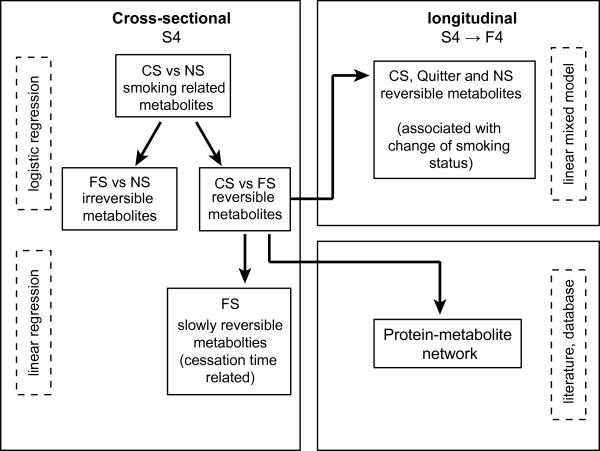
**Flow diagram illustrating the analysis strategy**. CS: current smokers; FS: former smokers; NS: never smokers.

Differences in population characteristics (CS, FS and NS) were tested by a two-tailed student's *t*-test. The metabolite concentrations were log transformed for normalization. We tested cross-sectional association of each metabolite with smoking using logistic regression models adjusted for age, body mass index (BMI) and alcohol consumption (see Figure 1). To correct for multiple testing, false discovery rate (FDR) was calculated using the Benjamini-Hochberg method [34] and the cut-off for statistical significance was set at FDR <0.05.

Linear regression models were used to investigate whether smoking intensities measured in pack years and cessation time are associated with metabolite concentrations. In the case of CS, the years of smoking were calculated as the time period from starting smoking until the start of the survey. Pack year was calculated as the number of cigarettes per day multiplied by smoking duration and divided by 20 [35]. Cessation time (in years) was calculated according to the questionnaire. The models contained the log-transformed metabolite concentrations as the dependent variable and the smoking intensities as the explanatory variable, with age, BMI and alcohol consumption as covariates. Every unit change of one covariate corresponds to a relative change of the metabolite concentration by Δ (%):

$$\text{Δ}=\left(\text{exp}\left({\text{β}}_{i}\right)-1\right)\times \mathrm{100}\%$$

where β_i _indicates the estimate of *ith *covariate in the model.

To assess the role of smoking cessation for the quitters, who were CS at S4 but FS at F4, we fitted the linear mixed models to the longitudinal data of KORA S4 → F4. The models contained the fixed effect of smoking status (CS, FS and NS), age, BMI and alcohol consumption with a random effect assigned to each participant. All calculations were performed in R (version 2.14.1).

### Network and pathway analysis

We retrieved protein-protein interactions from the databases of the Search Tool for the Retrieval of Interacting Genes/Proteins [36] and the relationships between enzymes and metabolites from the Human Metabolome Database [37] to construct protein-metabolite networks containing links between metabolites, enzymes and smoking-related genes. Genes and metabolites were connected allowing for at most one intermediate enzyme by Dijkstra's algorithm [38], and optimized by eliminating edges with Search Tool for the Retrieval of Interacting Genes/Proteins scores less than 0.7. Each edge in the networks was manually checked. We have implemented this method in our previous studies [29,39]. The analysis was performed using the R package igraph [40]. The network was visualized using Cytoscape [41]. Pathway analysis was performed by MetaboAnalyst [42].

## Results

### Characteristics of participants of the cross-sectional KORA S4

Participants were divided into three groups according to their self-reported smoking status. Population characteristics are shown in Table 1. On average, CS were two to three years younger and had a lower BMI than FS and NS. Male CS showed higher alcohol consumption than male NS, but there was no significant difference observed in women. Furthermore, the statistics showed differences in lifestyle factors between men and women. Alcohol consumption was higher in men than women (*P *= 1.5e^-11 ^(CS); *P *= 2.2e^-18 ^(FS); *P *= 9.5e^-17 ^(NS)), and smoking intensity (in pack years) was higher in male than in female CS (*P *= 6.0e^-6^).

**Table 1:** Characteristics of cross-sectional KORA S4.

	Current smoker	Former smoker	Never smoker	*P*^a^	
				
				Current versus former smoker	Current versus never smoker
**Male (N = 646)**					
N (%)	125 (19.3%)	321 (49.7%)	200 (31.0%)		
Age (years)	62.2 ±5.3	65.3 ± 5.3	64.1 ± 5.6	7.9e^-08^	3.0e^-03^
BMI (kg/m^2^)	27.0 ±3.6	28.9 ±3.6	27.8 ±3.4	1.5e^-06^	6.5e^-02^
Alcohol consumption (g/day)	27.5 ±29.0	24.1 ±24.3	20.5 ±21.3	0.25	0.02
Pack years^b^	39.3 ±22.4				
Quit time^c ^(years)		23.6 ±12.6			
**Female (N = 595)**					
N (%)	70 (11.8%)	130 (21.8%)	395 (66.4%)		
Age (years)	61.3 ±5.2	64.0 ±5.2	64.6 ±5.3	7.5e^-04^	5.9e^-06^
BMI (kg/m^2^)	27.2 ±4.5	28.7 ±5.0	28.5 ±4.6	0.029	0.02
Alcohol consumption (g/day)	6.5 ±10.9	10.0 ±12.8	7.5 ±11.1	0.042	0.48
Pack years^b^	25.8 ±15.3				
Quit time^c ^(years)		20.9 ±13.1			

The study characteristics of KORA S4 are shown separately for current, former and never smokers. Values are shown as mean ±SD when appropriate. ^a^*P*-values are calculated by student's *t*-test; ^b^calculated as the number of cigarettes consumed per day × years of smoking/20; ^c ^the time till the survey is conducted since the person has stopped smoking. BMI: body mass index.

### Metabolomic differences between current, former and never smokers

We identified 18 metabolites in men and six in women that were significantly different (FDR <0.05) between CS and NS. Three metabolites (PC ae C34:3, PC aa C36:1 and glutamate) were identified in both men and women showing the same pattern of variation (higher or lower) (Table 2). Compared with FS and NS, in male CS the concentrations of four unsaturated diacyl-PCs (PC aa C34:1, PC aa C36:1, PC aa C38:3 and PC aa C40:4) and five amino acids (arginine, aspartate, glutamate, ornithine and serine) were higher, whereas three saturated diacyl-PCs, one lysoPC and four acyl-alkyl-PCs, as well as kynurenine showed lower concentrations. In female CS, we found higher levels of carnitine and PC aa C32:1, and a lower level of hydroxysphingomyeline (SM (OH)) C22:2.

**Table 2:** Smoking-related metabolites in KORA S4.

Metabolites	CS versus NS		CS versus FS		FS versus NS	
			
	Odds ratio (95% CI)	*P*	Odds ratio (95% CI)	*P*	Odds ratio (95% CI)	*P *
		
Men	(125 versus 200)		(125 versus 321)		(321 versus 200)	
Arginine	1.7 (1.3, 2.2)	2.6e^-05a^	1.3 (1.0, 1.6)	0.03^a^	1.2 (1.0, 1.5)	0.03
Aspartate	1.6 (1.2, 2.0)	2.5e^-04a^	1.4 (1.1, 1.7)	4.7e^-03a^	1.1 (0.9, 1.3)	0.36
**Glutamate**	1.6 (1.2, 2.0)	6.2e^-04a^	1.4 (1.1, 1.9)	0.02^a^	1.0 (0.8, 1.3)	0.88
Ornithine	1.4 (1.2, 1.9)	2.2e^-03a^	1.3 (1.1, 1.7)	8.3e^-03a^	1.0 (0.9, 1.2)	0.78
Serine	1.4 (1.1, 1.8)	3.5e^-03a^	1.2 (1.0, 1.5)	0.12	1.1 (0.9, 1.4)	0.25
Kynurenine	0.6 (0.5, 0.9)	3.2e^-03a^	0.7 (0.5, 0.9)	2.3e^-03a^	1.0 (0.8, 1.2)	0.88
PC aa C32:3	0.7 (0.5, 0.9)	6.4e^-03a^	0.8 (0.6, 1.0)	0.07	0.9 (0.7, 1.0)	0.12
PC aa C34:1	1.7 (1.3, 2.2)	2.0e^-04a^	1.7 (1.3, 2.2)	2.5e^-05a^	0.9 (0.8, 1.1)	0.49
PC aa C36:0	0.6 (0.5, 0.8)	3.5e^-04a^	0.6 (0.5, 0.8)	2.7e^-04a^	1.0 (0.8, 1.2)	0.72
**PC aa C36:1**	1.6 (1.2, 2.0)	9.4e^-04a^	1.6 (1.3, 2.0)	8.2e^-05a^	0.9 (0.8, 1.1)	0.33
PC aa C38:0	0.7 (0.5, 0.9)	2.1e^-03a^	0.6 (0.5, 0.8)	1.2e^-04a^	1.0 (0.9, 1.3)	0.64
PC aa C38:3	1.5 (1.1, 1.9)	3.4e^-03a^	1.3 (1.1, 1.7)	0.01^a^	1.0 (0.8, 1.2)	0.85
PC aa C40:4	1.5 (1.2, 2.0)	3.4e^-03a^	1.4 (1.1, 1.8)	3.6e^-03a^	1.0 (0.8, 1.2)	0.86
**PC ae C34:3**	0.5 (0.4, 0.7)	3.3e^-06a^	0.6 (0.5, 0.8)	6.0e^-05a^	0.9 (0.7, 1.1)	0.23
PC ae C38:0	0.7 (0.5, 0.9)	2.1e^-03a^	0.6 (0.5, 0.8)	6.7e^-04a^	1.0 (0.8, 1.2)	0.94
PC ae C38:6	0.7 (0.5, 0.9)	4.8e^-03a^	0.7 (0.5, 0.8)	6.6e^-04a^	1.0 (0.8, 1.2)	0.97
PC ae C40:6	0.6 (0.5, 0.8)	8.8e^-04a^	0.7 (0.5, 0.8)	8.9e^-04a^	0.9 (0.8, 1.1)	0.33
lysoPC a C18:2	0.7 (0.5, 0.9)	3.3e^-03a^	0.8 (0.6, 0.9)	0.046^a^	0.9 (0.7, 1.1)	0.23
		
**Women**	**(70 versus 395)**		**(70 versus 130)**		**(130 versus 395)**	
carnitine	1.8 (1.4, 2.4)	4.3e^-05a^	1.5 (1.1, 2.1)	0.01^a^	1.1 (0.9, 1.4)	0.32
**Glutamate**	1.7 (1.3, 2.2)	1.2e^-04a^	1.8 (1.3, 2.5)	1.1e^-03a^	0.9 (0.7, 1.1)	0.17
PC aa C32:1	1.5 (1.1, 1.9)	2.1e^-03a^	1.4 (1.0, 2.0)	0.03^a^	1.1 (0.9, 1.4)	0.24
**PC aa C36:1**	1.6 (1.2, 2.0)	1.1e^-03a^	1.5 (1.1, 2.0)	0.02^a^	1.0 (0.8, 1.2)	0.87
**PC ae C34:3**	0.6 (0.4, 0.8)	7.7e^-04a^	0.6 (0.4, 0.8)	2.5e^-03a^	1.0 (0.8, 1.2)	0.94
SM (OH) C22:2	0.6 (0.5, 0.8)	2.1e^-03a^	0.6 (0.4, 0.9)	4.9e^-03a^	0.9 (0.7, 1.1)	0.35

Results of pair wise comparison by logistic regression of metabolites on smoking status adjusted for age, body mass index and alcohol consumption. Men and women were analyzed separately. We present all results with a false discovery rate (FDR) below 0.05 (in the comparison between CS and NS, the FDR was calculated by *P*-value adjusted for all 140 metabolites; for CS versus FS and FS versus NS, the FDR was calculated by *P*-value adjusted for the number of metabolites significantly different between CS and NS). Smoking-related metabolites found in both men and women are in bold. aa: diacyl-; ae: acyl-alkyl-; CI: confidence interval; CS: current smokers; FS: former smokers; NS: never smokers; PC: phosphatidylcholine; lysoPC: acyl-phosphatidylcholine; SM (OH): hydroxysphingomyeline. ^a^FDR <0.05.

**Figure 2:**
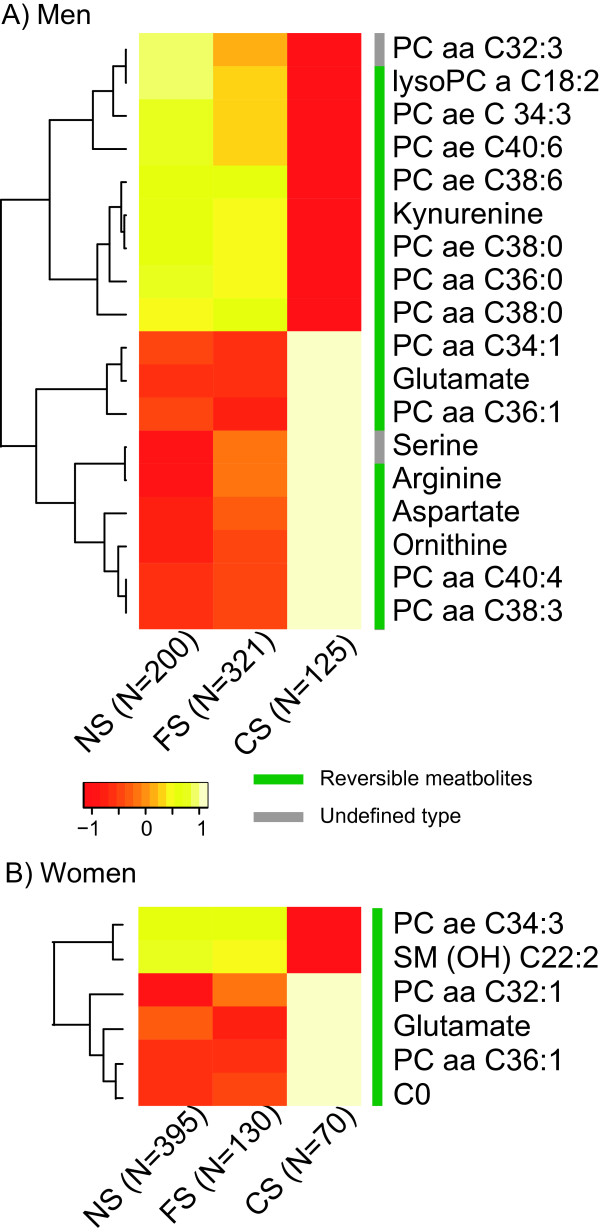
**Heat maps of smoking-related metabolites in (A) men and (B) women**. The heat map shows mean residues of smoking-related metabolites in CS, FS and NS and the reversibility after smoking cessation. The color of each cell in the heat map represents the relative mean concentration of each metabolite in NS, FS or CS. The number of samples in each group is provided. The bar besides the metabolite names indicates the reversibility of these metabolites after smoking cessation. aa: diacyl-; ae: acyl-alkyl-; C0: carnitine; CS: current smokers; FS: former smokers; lysoPC: acyl-phosphatidylcholine; NS: never smokers; PC: phosphatidylcholine; SM (OH): hydroxysphingomyeline.

Among the 21 smoking-related metabolites (18 in men and six in women), 19 were found to be reversible (that is, significant difference between FS and CS but without significant difference between FS and NS; FDR <0.05). No irreversible metabolite was observed (that is, significant difference between FS and NS). Serine and PC aa C32:3 in men were not classified because their concentrations were not significantly different between CS and FS or between FS and NS (Table 2). A heat map representing the concentration profiles of the 21 identified metabolites in CS, FS and NS is shown in Figure 2, demonstrating the reversibility of metabolites after smoking cessation.

**Figure 3:**
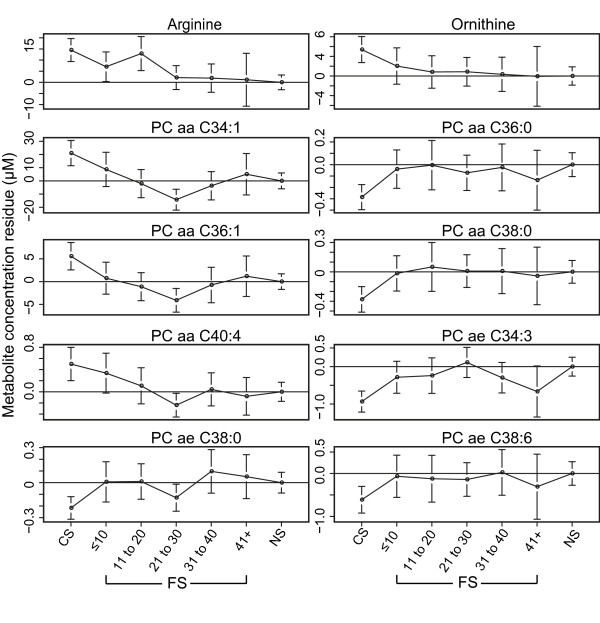
**Metabolite concentration variations in relation to smoking cessation time**. Taking NS as baseline, figures show the mean residuals of metabolites in different groups of CS and FS, giving the trend of metabolite variation with cessation time. FS were grouped by stratified cessation time (≤10, 11 to 20, 21 to 30, 31 to 40, 41+). Residuals were calculated by linear regression model (regression of metabolite concentration on age, body mass index and alcohol consumption). aa: diacyl-; ae: acyl-alkyl-; CS: current smokers; FS: former smokers; NS: never smokers; PC: phosphatidylcholine.

In women, SM (OH) C22:2 was significantly associated with cessation time (FDR <0.05); however, there was no such significant metabolite in men (Table S1 in Additional file 1), indicating a non-linear relationship between cessation time and the reversion of metabolite profile. In addition, we grouped the FS by stratified cessation years (0 to 10, 11 to 20, 21 to 30, 31 to 40, over 40 years). For some metabolites (for example, PC ae C38:0, PC aa C36:0 and ornithine), the greatest change of concentration occurred within the first 10 years of cessation compared with CS (Figure 3).

Within CS, we found kynurenine and PC ae C34:3, PC ae C38:0 and PC ae C38:6 in men, and PC aa C36:1 in women showing significant association with pack years. In the linear regression model, pack years showed a negative relation (parameter estimation β <0) to these five metabolites (Table 3) (for example, one pack year increase will lead to a decrease of the kynurenine level in CS by 0.33%).

**Table 3:** Smoking intensity (pack years) related to metabolites

Metabolites	β estimate of pack year	Δ (%)	*P*
	**(95% confidence interval)×10^-3^**		

**Men**			
Arginine	-1.1 (-3.6, 1.4)	-0.11%	0.38
Aspartate	2.9 (-1.4, 7.1)	0.29%	0.20
Glutamate	2.9 (-1.2, 6.9)	0.29%	0.17
Ornithine	-2.4 (-5.2, 0.3)	-0.24%	0.09
Serine	1.1 (-1.3, 3.6)	0.11%	0.37
Kynurenine*	-3.3 (-6.1, -0.5)	-0.33%	0.02
PC aa C32:3	-1.4 (-4.3, 1.4)	-0.14%	0.33
PC aa C34:1	-0.9 (-3.5, 1.6)	-0.09%	0.48
PC aa C36:0	-2.3 (-4.9, 0.4)	-0.23%	0.09
PC aa C36:1	-1.4 (-4.6, 1.8)	-0.14%	0.39
PC aa C38:0	-2.1 (-4.9, 0.7)	-0.21%	0.15
PC aa C38:3	1.2 (-1.7, 4.1)	0.12%	0.43
PC aa C40:4	1.3 (-2.5, 5.1)	0.13%	0.51
PC ae C34:3*	-3.7 (-6.4, -0.9)	-0.37%	0.01
PC ae C38:0*	-3.6 (-6.6, -0.5)	-0.36%	0.02
PC ae C38:6*	-2.6 (-5.1, -0.1)	-0.26%	0.04
PC ae C40:6	-1.7 (-4.4, 1.0)	-0.17%	0.22
lysoPC a C18:2	-3.1 (-6.5, 0.3)	-0.31%	0.07

**Women**			
Carnitine	1.1 (-4.3, 6.5)	0.11%	0.70
PC aa C32:1	0.2 (-10.5, 10.9)	0.02%	0.97
PC aa C36:1*	6.9 (0.6, 13.2)	0.69%	0.04
PC ae C34:3	-2.7 (-7.7, 2.2)	-0.27%	0.54
SM (OH) C22:2	-2.8 (-7.7, 2.2)	-0.28%	0.28
Glutamate	2.2 (-7.8, 12.2)	0.22%	0.67

Results of linear regression of smoking intensity (pack years) on metabolite concentrations in men and women, adjusted for age, body mass index and alcohol consumption. All smoking-related metabolites presented in Table 2 are listed (*P <0.05). aa: diacyl-; ae: acyl-alkyl-; CS: current smokers; FS: former smokers; lysoPC: acyl-phosphatidylcholine; NS: never smokers; PC: phosphatidylcholine; SM (OH): hydroxysphingomyeline.

### Prospective change of metabolite profiles (from KORA baseline S4 to follow-up F4)

**Figure 4:**
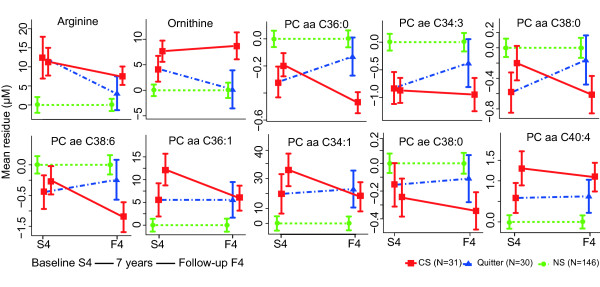
**Changes of smoking-related metabolites in current, former and never smokers in KORA S4 → F4**. Taking the NS as baseline, the concentration change of each metabolite is shown as the adjusted mean residue in KORA S4 and F4 in all three groups (CS, FS and NS). Only metabolites with significant prospective change in KORA S4 **→ **F4 are shown in the figure. Residuals were calculated from a linear regression model (regression of metabolite concentration on age, body mass index and alcohol consumption). aa: diacyl-; ae: acyl-alkyl-; CS: current smokers; FS: former smokers; lysoPC: acyl-phosphatidylcholine; NS: never smokers; PC: phosphatidylcholine.

The prospective dataset included 40 CS, 432 NS and 49 quitters (people who were CS in KORA S4 but FS in KORA F4) (Table 4). Among the 16 reversible metabolites in men, 13 (except kynurenine, glutamate and aspartate) were also measured in KORA F4 using a different kit (see Methods). We employed a linear mixed effect model to investigate the effects of smoking cessation on metabolite concentrations. Among these 13 metabolites, 10 metabolites showed a significant variation in quitters, with a period of smoking cessation from one to seven years, which indicated a reverting process. The arginine level decreased by 11.3% and ornithine by 14.8% in quitters compared with CS, whereas PC aa C36:0 increased by 18.5%. Figure 4 shows the prospective changes of the significant metabolites. For women, the same analysis was conducted. Because the number of female quitters was small (N = 10), five metabolites that were measured in both KORA S4 and F4 showed borderline significance (*P *<0.05). However, none of these metabolites was found to be significant considering FDR <0.05 (see Table 5).

**Table 4:** Characteristics of the prospective dataset (KORA S4 → F4).

	Current smoker	Former smoker	Never smoker
**Men (N = 207)**			
N (%)	31 (15.0%)	30 (14.5%)	146 (70.5%)
Age at S4 (years)	60.2 ±5.3	63.0 ±5.0	63.0 ±5.5
Alcohol consumption (S4/F4)(g/day)	27.7 ±28.2/20.4 ±28.7	29.6 ±31.6/19.3 ±21.1	22.2 ±22.8/20.2 ±19.5
BMI (S4/F4) (kg/m^2^)	26.8 ±2.9/26.9 ±3.3	28.5 ±3.8/28.9 ±3.9	27.6 ±3.3/27.8 ±3.4

**Women (N = 314)**			
N (%)	18 (5.7%)	10 (3.2%)	286 (91.1%)
Age at S4	61.0 ±5.1	59.5 ±3.1	63.6 ±5.1
Alcohol consumption (S4/F4)(g/day)	7.6 ±11.6/7.4 ±11.8	4.7 ±6.7/10.7 ±14.1	7.6 ±11.2/7.3 ±11.4
BMI (S4/F4) (kg/m^2^)	27.9 ±5.1/27.7 ±5.3	26.9 ±3.9/27.4 ±5.1	28.6 ±4.5/28.9 ±4.7

Population characteristics were calculated based on 207 men and 314 women who participated in both the KORA S4 and F4 study. Values are provided as mean ± SD. BMI: body mass index.

**Table 5:** Association of reversible metabolites with smoking status change in the prospective dataset (KORA S4 → F4)

	β estimate of smoking status (95% confidence interval)	*P *
**Men**		
Arginine	-0.12 (-0.18, -0.06)	1.4e^-04a^
Ornithine	-0.16 (-0.24, -0.08)	2.1e^-04a^
PC aa C34:1	-0.09 (-0.15, -0.03)	3.3e^-03a^
PC aa C36:0	0.17 (0.09, 0.25)	6.4e^-05a^
PC aa C36:1	-0.12 (-0.18, -0.05)	8.5e^-04a^
PC aa C38:0	0.14 (0.06, 0.22)	3.0e^-04a^
PC aa C38:3	-0.04 (-0.11, 0.02)	1.7e^-01^
PC aa C40:4	-0.11 (-0.18, -0.03)	6.0e^-03^
PC ae C34:3	0.14 (0.06, 0.21)	3.5e^-04a^
PC ae C38:0	0.13 (0.05, 0.21)	1.8e^-03a^
PC ae C38:6	0.11 (0.04, 0.18)	1.5e^-03a^
PC ae C40:6	0.08 (0.01, 0.15)	2.1e^-02^
lysoPC a C18:2	0.03 (-0.06, 0.11)	5.2e^-01^

**Women**		
Carnitine	-0.12 (-0.20, -0.05)	1.4e^-03^
PC aa C32:1	-0.18 (-0.32, -0.03)	2.1e^-03^
PC aa C36:1	-0.11 (-0.20, -0.02)	2.0e^-02^
PC ae C34:3	0.09 (-0.02, 0.19)	0.95
SM (OH) C22.2	0.12 (0.02, 0.22)	1.9e^-02^

Result of smoking status on metabolite concentrations using linear mixed model for S4 → F4 longitudinal data, adjusted for age, BMI, and alcohol consumption. PC: phosphatidylcholine; aa: diacyl-; ae: acyl-alkyl-; lysoPC: acyl-phosphatidylcholine; SM (OH): hydroxysphingomyeline. ^a ^FDR<0.05.

### Smoking effects on metabolic network

**Figure 5:**
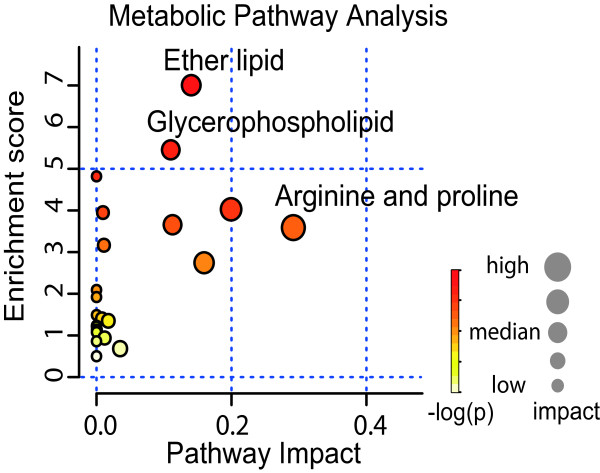
**Pathway analyses of smoking-related metabolites**. Figure shows enrichment and impact of smoking-related metabolites in Kyoto Encyclopedia of Genes and Genomes pathways. The enrichment scores are shown on y-axis, which was calculated as the negative logarithm of the *P*-value from an enrichment test. The x-axis indicates the structural impact with a score from 0 to 1 of the smoking-related metabolites in the enriched pathways.

Enrichment analysis of the 21 identified smoking-related metabolites on Kyoto Encyclopedia of Genes and Genomes pathways showed enrichment in a set of amino acid and lipid metabolism pathways (ether lipid, glycerophospholipid, arginine and proline metabolism). In addition, we analyzed the impact of the smoking-related metabolites in each pathway by measuring their structural importance (see Methods). These metabolites had high betweenness centrality and a strong impact on the enriched pathways (Figure 5 and Table S2 in Additional file 2).

**Figure 6:**
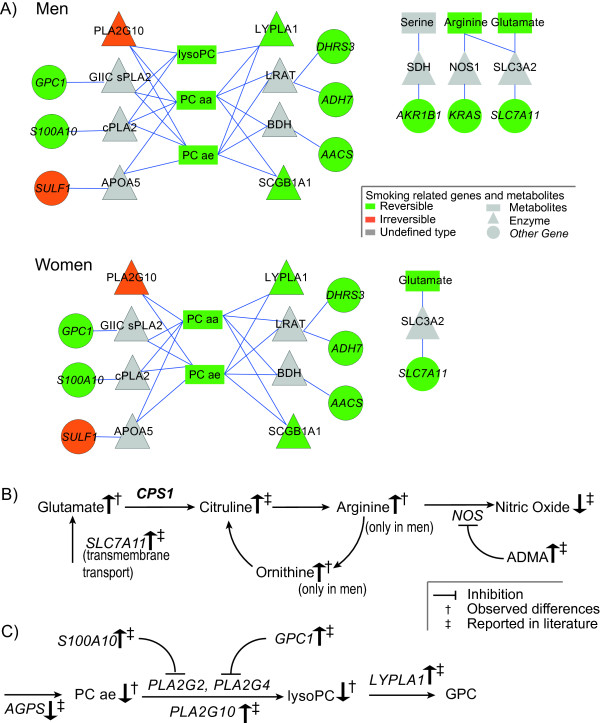
**Protein-metabolite networks and pathways of the smoking-related metabolites and genes**. **(A) **Network linking metabolites and proteins encoded by smoking-related genes with maximum one intermediate. Node color indicates the reversibility after smoking cessation. **(B, C) **Effects of smoking on arginine and glutamate as well as on lipid metabolism. Metabolites are in regular font, protein coding genes are in italic, gender-specific gene (CPS1) is in bold italic font. aa: diacyl-; ae: acyl-alkyl-; APOA5: apolipoprotein A-V; BDH: 3-hydroxybutyrate dehydrogenase, type 1; cPLA2: cytosolic phospholipase A2; CS: current smokers; FS: former smokers; GIIC sPLA2: phospholipase A2, membrane associated; LRAT: lecithin retinol acyltransferase; LYPLA1: lysophospholipase I; lysoPC: acyl-phosphatidylcholine; NOS1: nitric oxide synthase 1; NS: never smokers; PC: phosphatidylcholine; PLA2G10: group 10 secretory phospholipase A2; SCGB1A1: uteroglobin; SDH: serine dehydratase; SLC3A2: solute carrier family 3 member 2

To systematically investigate how the effects of smoking propagate over the metabolic networks, we evaluated the association between 175 smoking-related genes, previously reported [23], and the 21 smoking-related metabolites we found in this study by analyzing protein-metabolite networks (see Methods). In men, 15 metabolites (lysoPC a C18:2, PC aa C32:3,PC aa C34:1, PC aa C36:0, PC aa C36:1, PC aa C38:0, PC aa C38:3, PC aa C40:4, PC ae C34:3, PC ae C38:0, PC ae C38:6, PC ae C40:6, arginine, glutamate and serine) were found to be linked with 11 genes (*ADH7, AKR1B1, DHRS3, FTL, GALE, GPC1, KRAS, S100A10, SLC7A11, SULF1, PLA2G10*) by related enzymes. In women, four metabolites (PC aa C36:1, PC ae C34:3, PC aa C32:1 and glutamate) were closely linked with nine genes (*ADH7, AKR1B1, DHRS3, FTL, GALE, GPC1, S100A10, SULF1, PLA2G10*) (Figure 6A and Table S3 in Additional file 3). Similar to enrichment analysis, the network in men and in women could be generally divided into glycerophospholipids and tightly associated proteins as well as amino acids and the associated genes and enzymes. A description of the protein-metabolite and protein-protein interactions was listed in Table S3 in Additional file 3.

The smoking effects on the networks were reversible. With regards to gene expressions, with the exception of *SULF1 *and *PLA2G10*, all changes in the networks were reversible after smoking cessation [23]. All changes in metabolites in the network were also reversible, except serine.

## Discussion

In this study, we have used an 'omics' approach to investigate the association of metabolite concentrations with smoking, delineated the reversion of metabolite variations after smoking cessation and demonstrated the results using protein-metabolite networks. We identified strong associations of various metabolites with smoking, and confirmed part of the findings of our pilot study [1]. Among the 23 smoking-related metabolites identified in the pilot study, 11 metabolites were measured in this study, five of which (four unsaturated diacyl-PCs and one acyl-alkyl-PC) were validated in men, based on about five-fold larger CS samples. Consistent patterns of smoking effects on metabolite profile were observed in the current study. Among all the smoking-related metabolites, in CS we found higher unsaturated diacyl-PCs, but lower acyl-alkyl-PCs and saturated diacyl-PCs, which may indicate generally increased levels of unsaturated fatty acids in CS. Unsaturated fatty acids are more vulnerable to lipid peroxidation and influence the risk of different diseases [43,44].

### Smoking-related metabolites and cardiovascular disease

The study results implied the potential of metabolomics in revealing the role of an environmental factor, for example a smoking lifestyle, in the pathogenesis and prognosis of CVD.

One study on the peripheral blood metabolite profile showed an association of coronary artery disease and urea cycle-related metabolites, including arginine and glutamate [45], which were also identified in our study as smoking-related metabolites. By scrutinizing the smoking-related metabolites in metabolic pathways, we found further support for the pathophysiological relation between these metabolites and CVD. Previous findings indicated that the glutamate transporter in human lung epithelial cells, encoded by the *SLC7A11 *gene, is activated in CS [23,46], which increases the transportation of glutamate and subsequently raises the levels of the downstream metabolites, arginine and ornithine (Figure 6B). The activation of the cysteine-glutamate transporter (encoded by *SLC7A11*) and the increased glutamate level as a response to oxidative stress is also of great importance to endothelial dysfunction involved at all stages of atherosclerotic plaque evolution, which leads to CVD [47,48].

Ether lipid and glycerophospholipid metabolisms are associated with smoking [1,49]. The decreased level of lysoPC a C18:2 reflects the inhibition of upstream synthesis and activation of downstream hydrolysis. As shown in Figure 6C, upregulation of *S100A10 *and *GPC1 *inhibits cytosolic phospholipase A2, which plays a role in the synthesis of lyso-PCs. The lysophospholipase I isoform, which hydrolyses lysoPC into glycerophosphocholine, is upregulated in CS [23]. Interestingly, one recent study showed that a disorder of phosphatidylcholine metabolism would promote CVD [50], which may establish a link between smoking-related phosphatidylcholine variation and cardiovascular events. For example, the phosphatidylcholine hydroperoxide will promote angiogenesis in endothelial cells that are associated with atherosclerotic development [51].

The reversibility of metabolite concentrations in a small time window may reveal a reduced risk of smoking-related diseases after stopping smoking. Concentrations of arginine and glutamate that are associated with both smoking and coronary artery diseases quickly returned to normal levels (within seven years) after smoking cessation, which is in line with epidemiological findings that the smoking effects on CVD are quickly and largely reduced after smoking cessation [8,9,52]. The reversed glutamate level indicates reduced oxidative stress after smoking cessation, and the reversion of arginine and ornithine reflects a reversion of functioning in the urea cycle. Our findings provide metabolic insight into the reduced risk of CVD after smoking cessation and provide support for the remarkable benefits people would gain by stopping smoking.

### Concordance of reversibility in metabolic network

The protein-metabolite interaction network shows that the reversibility of metabolite concentrations also coincided with gene expression (Figure 6A). Arginine and glutamate were quickly reversed after smoking cessation, which was in line with the quick reversibility of *SLC7A11 *expression. Expression of enzyme coding genes for the hydrolysis of diacyl-PCs and acyl-alkyl-PCs, for instance lysophospholipase, cytosolic phospholipase A2 and S100 calcium binding protein A2, were quickly reversible and smoking-related diacyl-PCs and acyl-alkyl PCs shared the same reverse pattern.

### Gender-specific effects of smoking

In this study, we found gender-specific effects of smoking on metabolite profiles (Table S1 in Additional file 1). This result supports the assumption that differences in smoking effects on men and women are not solely based on smoking intensity but are also gender-specific. Glutamate was higher in both male and female CS, however, the levels of arginine and ornithine were only higher in male CS. According to a previous study of the metabolomic and genetic biomarkers on sexual dimorphisms [30], the *CPS1 *gene, which regulates the formation of arginine, has a gender-specific manner in certain single nucleotide polymorphisms, with stronger effects in women than in men. The gender-specific genetic effect might cause a lower efficiency in women in regard to the transformation of extra glutamate to citrulline (Figure 6C).

### Strengths and limitations

We used a systematic targeted metabolomics approach with 140 metabolites in a large population-based cohort. Analyzing the effects of smoking and smoking cessation in this prospective manner (follow-up of seven years) provides more power to investigate smoking effects by ruling out individual differences. However, our study is based on a limited range and number of metabolites and cannot fully represent the whole metabolome. Thus, an improved metabolomics technique measuring more metabolites is urgently needed for a comprehensive understanding of both reversible and permanent effects of smoking on human metabolism. It would be interesting for future studies to also include data on other environmental factors such as diet and lifestyle, which are known to have effects on the human metabolome [53,54].

## Conclusions

Our study shows the power of the metabolomics approach in investigating the molecular signature of lifestyle-related environmental exposures. We demonstrated that smoking is associated with concentration variations in amino acids, ether lipid and glycerophospholipid metabolism at an 'omics' level. The smoking-related changes in the human serum metabolite profile are reversible after stopping smoking. This indicates the remarkable benefits of smoking cessation and provides a link to CVD benefits. Furthermore, linking metabolomic knowledge to other 'omics' approaches, for example, transcriptomics, may have the potential to identify novel biomarkers as well as new risk assessment tools.

## Abbreviations

aa: diacyl-; ae: acyl-alkyl-; BMI: body mass index; CS: current smokers; CVD: cardiovascular disease; FDR: false discovery rate; FS: former smokers; lysoPC: acyl-phosphatidylcholine; NS: never smokers; PC: phosphatidylcholine; SM: sphingomyeline; SM (OH): hydroxysphingomyeline.

## Competing interests

The authors declare that they have no competing interests.

## Authors' contributions

HEW, KS, JA, TI, AP and RWS initiated and designed the study. CP, WRM, WR, HEW, KHL and JA were involved in and performed the experiment. TX and ZY performed the data analysis. TX, CH, ZY and RWS wrote the manuscript, XD, EB, CP, KP, MJ, YL, HW, FT, JA and AP revised the manuscript. The manuscript has been approved by all authors.

## Pre-publication history

The pre-publication history for this paper can be accessed here:

http://www.biomedcentral.com/1741-7015/11/60/prepub

## Supplementary Material

Additional file 1 Table S1: Cessation time-related metabolites in FS. FDR was calculated by *P*-value adjusted for the number of smoking-related metabolites with Benjamini-Hochberg method. aa: diacyl-; ae: acyl-alkyl-; C0: carnitine; FS: former smokers; lysoPC: acyl-phosphatidylcholine; PC: phosphatidylcholine; SM (OH): hydroxysphingomyeline. Click here for file

Additional file 2 **Table S2: Enrichment and impact of smoking-related metabolites in Kyoto Encyclopedia of Genes and Genomes pathways**. Table shows the enrichment and impact scores of smoking-related metabolites in Kyoto encyclopedia of Genes and Genomes pathways. The pathway analysis consists of enrichment and a structural impact analysis both based on Kyoto Encyclopedia of Genes and Genomes database. The -log (*P*) was considered as the enrichment score. Impact, scored between 0 and 1, indicated the pathway topological importance of the metabolites. In particular, the parameter Total is the total number of compounds in the pathway; the parameter Hits is the actual number of metabolites with significant variations in the pathway; the Raw *P *was the original *P*-value calculated from the enrichment analysis; the FDR was calculated as the *P*-value adjusted using Benjamini-Hochberg method. Click here for file

Additional file 3 **Table S3: Links between smoking-related metabolites, enzymes and genes**. The table describes the links showed in Figure 6 of the main text. The smoking-related metabolites, enzymes and genes are listed in the first and second columns. The score of interaction is given according to the definition by the Search Tool for the Retrieval of Interacting Genes/Proteins [1]. A reference for each link and a short description is provided. The Column of reaction shows the possible biochemical reaction of the corresponding link or the type of protein interaction. The enzymes includes, phospholipase A2, membrane associated (GIIC sPLA2), cytosolic phospholipase A2 (cPLA2), group 10 secretory phospholipase A2 (PLA2G10), lysophospholipase I (LYPLA1), apolipoprotein A-V (APOA5), uteroglobin (SCGB1A1), lecithin retinol acyltransferase (LRAT), nitric oxide synthase 1 (NOS1), solute carrier family 3 member 2 (SLC3A2), serine dehydratase (SDH), 3-hydroxybutyrate dehydrogenase, type 1 (BDH). The smoking-related gene/protein includes, S100 calcium binding protein A10 (*S100A10*), glypican 1 (*GPC1*), sulfatase 1 (*SULF1*), alcohol dehydrogenase 7 (*ADH7*), dehydrogenase member 3 (*DHRS3*), aldose reductase (*AKR1B1*), acetoacetyl-CoA synthetase (*AACS*), V-Ki-ras2 Kirsten rat sarcoma viral oncogene homolog (*KRAS*), solute carrier family 7 (*SLC7A11*) and three enzyme listed above, PLA2G10, LYPLA1, SCGB1A1. The links in the network for male and female CS are combined and listed together. Smoking-related genes are show in italic. aa: diacyl-; ae: acyl-alkyl-; C0: carnitine; lysoPC: acyl-phosphatidylcholine; PC: phosphatidylcholine; SM (OH): hydroxysphingomyeline. Click here for file
