# Supplementary material for: Effects of smoking and smoking cessation on human serum metabolite profile: results from the KORA cohort study
Source: BMC Med. 2013 Mar 4;11:60. doi: 10.1186/1741-7015-11-60 (PMC3653729; doi:10.1186/1741-7015-11-60)
Supplement: Additional file 1 — Table S1: Cessation time-related metabolites in FS. FDR was calculated by P-value adjusted for the number of smoking-related metabolites with Benjamini-Hochberg method. aa: diacyl-; ae: acyl-alkyl-; C0: carnitine; FS: former smokers; lysoPC: acyl-phosphatidylcholine; PC: phosphatidylcholine; SM (OH): hydroxysphingomyeline. [file 1741-7015-11-60-S1.DOC]

### Additional file 1 – Table S1 Cessation time related metabolites in FS

FDR was calculated by p-value adjusted for the number of smoking-related metabolites with Benjamini & Hochberg method. FS: former smokers; C0: carnitine; PC: phosphatidylcholine; aa: diacyl-; ae: acyl-alkyl-; lysoPC: acyl-phosphatidylcholine; SM (OH): hydroxysphingomyeline

| **Metabolites** | **β Estimate (95% CI)*10^-3^** | **Pr(>|t|)** | **FDR** |
| --- | --- | --- | --- |
| **Men** |  |  |  |
| Arginine | -1.7 (-4.2,0.8) | 0.19 | 0.43 |
| Asparate | -3.4 (-7.5,0.7) | 0.1 | 0.32 |
| Glutamate | -3.9 (-8.4,0.5) | 0.09 | 0.48 |
| Ornithine | -1.3 (-4.1,1.4) | 0.34 | 0.49 |
| Kynurenine | -0.2 (-3.0,2.6) | 0.87 | 0.93 |
| PC aa C34:1 | -0.4 (-2.8,2.0) | 0.72 | 0.89 |
| PC aa C36:0 | 1.9 (-1.2,5.0) | 0.24 | 0.43 |
| PC aa C36:1 | -0.4 (-3.3,2.5) | 0.77 | 0.88 |
| PC aa C38:0 | 2.0 (-1.1,5.1) | 0.21 | 0.42 |
| PC aa C38:3 | -1.3 (-4.1,1.4) | 0.35 | 0.47 |
| PC aa C40:4 | -2.8 (-6.0,0.5) | 0.10 | 0.32 |
| PC ae C34:3 | 0.1 (-3.2,3.4) | 0.97 | 0.97 |
| PC ae C38:0 | 3.0 (-0.4,6.4) | 0.08 | 0.64 |
| PC ae C38:6 | 1.6 (-1.2,4.4) | 0.27 | 0.43 |
| PC ae C40:6 | 3.0 (0.3,5.7) | 0.03 | 0.48 |
| lysoPC a C18:2 | 2.6 (-0.8,6.1) | 0.14 | 0.37 |
| **Women** |  |  |  |
| C0 | 1.0 (-3.7,5.8) | 0.66 | 0.66 |
| PC aa C32:1 | -6.1 (-13.7,1.6) | 0.12 | 0.31 |
| PC aa C36:1 | -2.1 (-7.5,3.3) | 0.44 | 0.52 |
| PC ae C34:3 | 2.2 (-2.9,7.2) | 0.41 | 0.68 |
| SM OH C22:2***** | 5.4 (1.8,9.0) | 3.70E-03 | 0.02 |
| Glutamate | 3.1 (-4.6,10.8) | 0.43 | 0.64 |
