# Supplementary material for: Effects of smoking and smoking cessation on human serum metabolite profile: results from the KORA cohort study
Source: BMC Med. 2013 Mar 4;11:60. doi: 10.1186/1741-7015-11-60 (PMC3653729; doi:10.1186/1741-7015-11-60)
Supplement: Additional file 2 — Table S2: Enrichment and impact of smoking-related metabolites in Kyoto Encyclopedia of Genes and Genomes pathways. Table shows the enrichment and impact scores of smoking-related metabolites in Kyoto encyclopedia of Genes and Genomes pathways. The pathway analysis consists of enrichment and a structural impact analysis both based on Kyoto Encyclopedia of Genes and Genomes database. The -log (P) was considered as the enrichment score. Impact, scored between 0 and 1, indicated the pathway topological importance of the metabolites. In particular, the parameter Total is the total number of compounds in the pathway; the parameter Hits is the actual number of metabolites with significant variations in the pathway; the Raw P was the original P-value calculated from the enrichment analysis; the FDR was calculated as the P-value adjusted using Benjamini-Hochberg method. [file 1741-7015-11-60-S2.DOC]

### Additional file 2– Table S2 Enrichment and impact of smoking-related metabolites in KEGG pathways

Table shows the enrichment and impact scores of smoking-related metabolites in KEGG pathways. The pathway analysis consists of enrichment and a structural impact analysis both based on KEGG database. The -log (p) was considered as the enrichment score. Impact, scored between 0 and 1, indicated the pathway topological importance of the metabolites. In particular, the parameter Total is the total number of compounds in the pathway; the parameter Hits is the actually number of metabolites with significant variations in the pathway; the Raw p is the original p value calculated from the enrichment analysis; the FDR are calculated by the p values adjusted using Benjamini-Hochberg method.

|  | **Total** | **Hits** | **Raw p** | **-log(p)** | **FDR** | **Impact** |
| --- | --- | --- | --- | --- | --- | --- |
| Ether lipid metabolism | 23 | 3 | 9.1E-04 | 7.01 | 0.04 | 0.14 |
| Glycerophospholipid metabolism | 39 | 3 | 4.3E-03 | 5.45 | 0.09 | 0.11 |
| Cyanoamino acid metabolism | 16 | 2 | 0.01 | 4.82 | 0.13 | 0 |
| Alanine, aspartate and glutamate metabolism | 24 | 2 | 0.02 | 4.03 | 0.22 | 0.20 |
| Sphingolipid metabolism | 25 | 2 | 0.02 | 3.95 | 0.22 | 0.01 |
| Aminoacyl-tRNA biosynthesis | 75 | 3 | 0.03 | 3.66 | 0.25 | 0.11 |
| Arginine and proline metabolism | 77 | 3 | 0.03 | 3.59 | 0.25 | 0.29 |
| Glutathione metabolism | 38 | 2 | 0.04 | 3.17 | 0.34 | 0.01 |
| Glycine, serine and threonine metabolism | 48 | 2 | 0.06 | 2.74 | 0.47 | 0.16 |
| Linoleic acid metabolism | 15 | 1 | 0.12 | 2.09 | 0.82 | 0 |
| Sulfur metabolism | 18 | 1 | 0.15 | 1.92 | 0.90 | 0 |
| alpha-Linolenic acid metabolism | 29 | 1 | 0.23 | 1.49 | 1 | 0 |
| Vitamin B6 metabolism | 32 | 1 | 0.25 | 1.40 | 1 | 0.01 |
| Methane metabolism | 34 | 1 | 0.26 | 1.35 | 1 | 0.02 |
| Nitrogen metabolism | 39 | 1 | 0.29 | 1.23 | 1 | 0 |
| Butanoate metabolism | 40 | 1 | 0.30 | 1.21 | 1 | 0 |
| Histidine metabolism | 44 | 1 | 0.32 | 1.13 | 1 | 0.00 |
| Lysine degradation | 47 | 1 | 0.34 | 1.08 | 1 | 0 |
| Cysteine and methionine metabolism | 56 | 1 | 0.39 | 0.94 | 1 | 0.01 |
| Arachidonic acid metabolism | 62 | 1 | 0.42 | 0.86 | 1 | 0 |
| Tryptophan metabolism | 79 | 1 | 0.51 | 0.68 | 1 | 0.03 |
| Porphyrin and chlorophyll metabolism | 104 | 1 | 0.61 | 0.50 | 1 | 0 |
