# Supplementary material for: Effects of smoking and smoking cessation on human serum metabolite profile: results from the KORA cohort study
Source: BMC Med. 2013 Mar 4;11:60. doi: 10.1186/1741-7015-11-60 (PMC3653729; doi:10.1186/1741-7015-11-60)
Supplement: Additional file 3 — Table S3: Links between smoking-related metabolites, enzymes and genes. The table describes the links showed in Figure 6 of the main text. The smoking-related metabolites, enzymes and genes are listed in the first and second columns. The score of interaction is given according to the definition by the Search Tool for the Retrieval of Interacting Genes/Proteins [1]. A reference for each link and a short description is provided. The Column of reaction shows the possible biochemical reaction of the corresponding link or the type of protein interaction. The enzymes includes, phospholipase A2, membrane associated (GIIC sPLA2), cytosolic phospholipase A2 (cPLA2), group 10 secretory phospholipase A2 (PLA2G10), lysophospholipase I (LYPLA1), apolipoprotein A-V (APOA5), uteroglobin (SCGB1A1), lecithin retinol acyltransferase (LRAT), nitric oxide synthase 1 (NOS1), solute carrier family 3 member 2 (SLC3A2), serine dehydratase (SDH), 3-hydroxybutyrate dehydrogenase, type 1 (BDH). The smoking-related gene/protein includes, S100 calcium binding protein A10 (S100A10), glypican 1 (GPC1), sulfatase 1 (SULF1), alcohol dehydrogenase 7 (ADH7), dehydrogenase member 3 (DHRS3), aldose reductase (AKR1B1), acetoacetyl-CoA synthetase (AACS), V-Ki-ras2 Kirsten rat sarcoma viral oncogene homolog (KRAS), solute carrier family 7 (SLC7A11) and three enzyme listed above, PLA2G10, LYPLA1, SCGB1A1. The links in the network for male and female CS are combined and listed together. Smoking-related genes are show in italic. aa: diacyl-; ae: acyl-alkyl-; C0: carnitine; lysoPC: acyl-phosphatidylcholine; PC: phosphatidylcholine; SM (OH): hydroxysphingomyeline. [file 1741-7015-11-60-S3.DOC]

### Additional file 3 – Table S3 Links between smoking-related metabolites, enzymes and genes

The table describes the links showed in figure 6 of the main text. The smoking related metabolites, enzymes and genes are listed in the first and second columns. The score of interaction is given according to the definition by STRING [1]. A reference for each link and a short description is provided. The Column of reaction shows the possible biochemical reaction of the corresponding link or the type of protein interaction. The enzymes includes, phospholipase A2, membrane associated (GIIC sPLA2), cytosolic phospholipase A2 (cPLA2), group 10 secretory phospholipase A2 (PLA2G10), lysophospholipase I (LYPLA1), apolipoprotein A-V (APOA5), uteroglobin (SCGB1A1), lecithin retinol acyltransferase (LRAT), nitric oxide synthase 1 (NOS1), solute carrier family 3 member 2 (SLC3A2), serine dehydratase (SDH), 3-hydroxybutyrate dehydrogenase, type 1 (BDH). The smoking related gene/protein includes, S100 calcium binding protein A10 (*S100A10*), glypican 1 (*GPC1*), sulfatase 1 (*SULF1*), alcohol dehydrogenase 7 (*ADH7*), dehydrogenase member 3 (*DHRS3*), aldose reductase (*AKR1B1*), acetoacetyl-CoA synthetase (*AACS*), V-Ki-ras2 Kirsten rat sarcoma viral oncogene homolog (*KRAS*), solute carrier family 7 (*SLC7A11*) and three enzyme listed above, PLA2G10, LYPLA1, SCGB1A1. The links in the network for male and female CS are combined and listed together. Smoking-related genes are show in italic. FDR was calculated by p-value adjusted for the number of smoking-related metabolites with Benjamini & Hochberg method. C0: carnitine; PC: phosphatidylcholine; aa: diacyl-; ae: acyl-alkyl-; lysoPC: acyl-phosphatidylcholine; SM (OH): hydroxysphingomyeline

| **Metabolites / Enzymes** | **Proteins/Smoking related gene** | **Score** | **Type** | **Description** | **Reaction** |
| --- | --- | --- | --- | --- | --- |
| Glutamate | SLC3A2 | 1 | catalytic | SLC7A11-mediated exchange of extracellular cysteine and cytosolic glutamate | transport |
| Arginine | SLC3A2 | 1 | catalytic | SLC7A7 (y+LAT1)-mediated exchange of extracellular leucine for cytosolic arginine | transport |
| Arginine | NOS1 | 1 | oxidoreductase | Arginine and proline metabolism | L-arginine + n NADPH + n H+ + m O2 = citrulline + nitric oxide + n NADP+ |
| PC aa/ae CX:Y, LysoPC a C18:2 | *PLA2G10* | 1 | phospholipase | Glycerophospholipid metabolism | phosphatidylcholine + H2O = 1-acylglycerophosphocholine + a carboxylate |
| PC aa/ae CX:Y, LysoPC a C18:2 | cPLA2 | 1 | phospholipase | Glycerophospholipid metabolism | phosphatidylcholine + H2O = 1-acylglycerophosphocholine + a carboxylate |
| PC aa/ae CX:Y, LysoPC a C18:2 | *PLA2G2A* | 1 | phospholipase | Glycerophospholipid metabolism | phosphatidylcholine + H2O = 1-acylglycerophosphocholine + a carboxylate |
| PC aa/ae CX:Y, LysoPC a C18:2 | *LYPLA1* | 1 | hydrolase | Hydrolyzes fatty acids from S-acylated cysteine residues in proteins such as trimeric G alpha proteins or HRAS. Has depalmitoylating activity and also low lysophospholipase activity | 2-lysophosphatidylcholine + H2O=glycerophosphocholine + a carboxylate |
| PC aa/ae CX:Y | APOA5 | 1 | lipid binding | Interact with phosphatidylcholine via lipoprotein lipase (LPL) |  |
| PC aa/ae CX:Y | LRAT | 1 | phosphatidylcholine-retinol O-acyltransfera | Transfers the acyl group from the sn-1 position of phosphatidylcholine to all-trans retinol, producing all-trans retinyl esters. Retinyl esters are storage forms of vitamin A. | phosphatidylcholine + retinol---[cellular-retinol-binding-protein] = 2-acylglycerophosphocholine + retinyl-ester---[cellular-retinol-binding-protein] |
| PC aa/ae CX:Y | *SCGB1A1* | 1 | binding | Binds phosphatidylcholine, potent inhibitor of phospholipase A2 |  |
| PC aa/ae CX:Y | BDH | 1 | activation | BDH activated by phosphotadylcholine |  |
| Serine | SDS | 1 | catalytic | Binding and dehydrate | L-threonine = 2-oxobutanoate + NH3 |
| SM OH C22:2 | *SGMS1* | 1 | catalytic | Bidirectional lipid cholinephosphotransferase capable of converting phosphatidylcholine (PC) and ceramide to sphingomyelin (SM) and diacylglycerol (DAG) and vice versa. | a ceramide + a phosphatidylcholine = a sphingomyelin + a 1,2-diacyl-sn-glycerol |
| *SLC7A11* | SLC3A2 | 0.99 | Binding | 1. SLC7A11:SLC3A2 heterodimer; SLC7A11-mediated exchange of extracellular cysteine and cytosolic glutamate.  2. In vivo Experimental data |  |
| *KRAS* | NOS1 | 0.83 | same pathway | Long-term depression |  |
| GIIC sPLA2 | *GPC1* | 0.92 | binding | Inferred from physical interaction |  |
| cPLA2 | *S100A10* | 0.83 | Inhibition | The antiinflammatory protein annexin-1 (ANXA1) and the adaptor S100A10 (p11), inhibit cytosolic phospholipase A2 (cPLA2alpha) by direct interaction. | S100A10 --| cPLA2 |
| APOA5 | *SULF1* | 0.72 | Binding | low-density lipoprotein receptor family and glycosylphosphatidylinositol high-density lipoprotein binding protein1. |  |
| BDH | *AACS* | 0.96 | same pathway | Butanoate metabolism | up and down stream |
| LRAT | *ADH7* | 0.9 | same pathway | Retinol metabolism | up and down stream |
| LRAT | *DHRS3* | 0.96 | same pathway | Retinol metabolism | up and down stream |
| SDH | *AKR1B1* | 0.8 | inter pathway | Inter-pathway connection between 'Glycine, serine and threonine metabolism' and 'Pyruvate metabolism' | up and down stream |

**Proteins/Enzymes with alternative abbreviations:**

PLA2G2A (GIIC sPLA2), Phospholipase A2, membrane associated"

PLA2G4A (cPLA2), Cytosolic phospholipase A2

BDH1 (BDH), D-beta-hydroxybutyrate dehydrogenase, mitochondrial

SDS (SDH), L-serine dehydratase

**Reference:**

1. Wu, G. and C.J. Meininger, *Arginine nutrition and cardiovascular function.* J Nutr, 2000. **130**(11): p. 2626-9.

2. Dijkstra, E.W., *A note on two problems in connexion with graphs.* Numerische Mathematik, 1959. **1**: p. 2.

3. Chen, H., et al., *Detrimental metabolic effects of combining long-term cigarette smoke exposure and high-fat diet in mice.* Am J Physiol Endocrinol Metab, 2007. **293**(6): p. E1564-71.

4. Kanehisa, M., et al., *The KEGG databases at GenomeNet.* Nucleic Acids Res, 2002. **30**(1): p. 42-6.

5. Wishart, D.S., et al., *HMDB: a knowledgebase for the human metabolome.* Nucleic Acids Res, 2009. **37**(Database issue): p. D603-10.

6. Wichmann, H.E., C. Gieger, and T. Illig, *KORA-gen--resource for population genetics, controls and a broad spectrum of disease phenotypes.* Gesundheitswesen, 2005. **67 Suppl 1**: p. S26-30.

7. Jahng, W.J., L.L. Xue, and R.R. Rando, *Lecithin retinol acyltransferase is a founder member of a novel family of enzymes.* Biochemistry, 2003. **42**(44): p. 12805-12812.

8. Shen, X.Z., et al., *Hormonal regulation of rabbit uteroglobin gene transcription.* Endocrinology, 1983. **112**(3): p. 871-6.

9. Tikkanen, M.J., et al., *Effects of oestradiol and levonorgestrel on lipoprotein lipids and postheparin plasma lipase activities in normolipoproteinaemic women.* Acta Endocrinol (Copenh), 1982. **99**(4): p. 630-5.

10. Demel, R.A., K. Shirai, and R.L. Jackson, *Lipoprotein lipase-catalyzed hydrolysis of tri[14C]oleoylglycerol in a phospholipid interface. A monolayer study.* Biochim Biophys Acta, 1982. **713**(3): p. 629-37.

11. Tandon, R.S. and K.C. Misra, *Threonine and serine dehydratase activity in the buffalo liver-fluke Fasciola indica.* J Helminthol, 1980. **54**(4): p. 259-62.

12. Tani, M. and O. Kuge, *Sphingomyelin synthase 2 is palmitoylated at the COOH-terminal tail, which is involved in its localization in plasma membranes.* Biochemical and Biophysical Research Communications, 2009. **381**(3): p. 328-332.

13. Kim, J.Y., et al., *Human cystine/glutamate transporter: cDNA cloning and upregulation by oxidative stress in glioma cells.* Biochimica Et Biophysica Acta-Biomembranes, 2001. **1512**(2): p. 335-344.

14. Yun, H.Y., et al., *Nitric oxide mediates N-methyl-D-aspartate receptor-induced activation of p21ras.* Proc Natl Acad Sci U S A, 1998. **95**(10): p. 5773-8.

15. Boilard, E., et al., *Interaction of low molecular weight group IIA phospholipase A2 with apoptotic human T cells: role of heparan sulfate proteoglycans.* FASEB J, 2003. **17**(9): p. 1068-80.

16. Borot, F., et al., *Eicosanoid Release Is Increased by Membrane Destabilization and CFTR Inhibition in Calu-3 Cells.* PLoS One, 2009. **4**(10).

17. Forte, T.M., X. Shu, and R.O. Ryan, *The ins (cell) and outs (plasma) of apolipoprotein A-V.* Journal of Lipid Research, 2009. **50**: p. S150-S155.

18. Ashla, A.A., et al., *Genetic analysis of expression profile involved in retinoid metabolism in non-alcoholic fatty liver disease.* Hepatol Res, 2010. **40**(6): p. 594-604.
